# Integrating Mathematics into Prenatal Diagnosis—Different Phenotypes of Complex Ventral Wall Malformations Determined by Hierarchical Clustering

**DOI:** 10.3390/jcm15041343

**Published:** 2026-02-08

**Authors:** Julia Bijok, Anna Kucińska-Chahwan, Diana Massalska, Marcin Bodziak, Tomasz Roszkowski

**Affiliations:** 1 Centre of Endoscopic Simulation, Centre of Postgraduate Medical Education, 00-416 Warsaw, Poland; 2 Department of Medical Genetics, Institute of Mother and Child, 01-211 Warsaw, Poland; 3 Department of Obstetrics and Gynecology, Institute of Mother and Child, 01-211 Warsaw, Poland; 4 IInd Department of Obstetrics and Gynecology, Centre of Postgraduate Medical Education, Warsaw Institute of Women’s Health, 00-189 Warsaw, Poland; diana_massalska@wp.pl; 5 Data Science Department, Accenture Research, 01-211 Warsaw, Poland

**Keywords:** cluster analysis, limb body wall complex, ventral wall, body stalk anomaly, acrania, neural tube defect, kyphoscoliosis, encephalocoele, pelvic wall defect, bladder exstrophy

## Abstract

**Background/Objectives:** To identify distinct sonographic phenotypes of complex malformations of the fetal ventral wall. **Methods:** We performed a retrospective analysis of ultrasound reports from 160 fetuses diagnosed with complex ventral wall defects at a single tertiary referral center between 1997 and 2021. Agglomerative hierarchical clustering was applied to identify distinct sonographic phenotypes based on the level of the ventral wall defect and associated anomalies. **Results:** Ventral wall defects involved the abdominal wall in 150 cases, the thoracic wall in 42 cases, and the pelvic wall in 28 cases, either in isolation or in combination. Open neural tube defects were present in 58 fetuses (36.3%), spinal defects in 110 fetuses (68.8%), and limb anomalies in 45 fetuses (28.1%). Additional anomalies were identified in 38 fetuses (23.8%), including cardiac anomalies in 18 cases (11.3%). Amniotic bands were observed in seven cases (4.4%). Using agglomerative hierarchical clustering, five groups of fetuses with differing numbers of observations were identified (cluster 1, *n* = 104; cluster 2, *n* = 5; cluster 3, *n* = 30; cluster 4, *n* = 10; cluster 5, *n* = 11). The silhouette score of the clustering model was 0.3285. The most discriminative features for each cluster, expressed as feature importance values, were as follows: kyphoscoliosis for cluster 1 (0.924), pelvic wall defect for cluster 2 (0.852), ectopia cordis for cluster 3 (0.662), limb anomalies for cluster 4 (0.767), and spina bifida for cluster 5 (0.691). **Conclusions:** Complex malformations of the fetal ventral wall are associated with a wide spectrum of additional anomalies. Hierarchical clustering identified five distinct sonographic phenotypes of complex ventral wall defects, highlighting the heterogeneity of these conditions.

## 1. Background and Aim of the Study

The formation of the ventral body wall occurs during the first four weeks of embryonic life, concomitantly with other processes of blastogenesis such as formation of the limbs, umbilical cord, and neural tube [1]. Consequently, malformations of the fetal body wall are frequently associated with a wide range of additional structural anomalies [2]. Several phenotypes of complex body wall malformations have been described, including limb body wall complex (LBWC), body stalk anomaly (BSA), amniotic band syndrome (ABS), pentalogy of Cantrell, and the omphalocele–exstrophy of the bladder–imperforate anus–spinal defects (OEIS) complex [3,4,5,6,7,8,9].

Due to substantial overlap in phenotypic features, the definitions and diagnostic criteria for these conditions have often been used interchangeably, with variable inclusion criteria, leading to inconsistency and confusion in the literature. In particular, variations in the clinical presentation of LBWC have resulted in multiple proposed classification systems [4,5,10,11]. However, most existing classifications are based primarily on postmortem findings, which are not always directly applicable to the prenatal period. For example, amniotic bands are reported less frequently on prenatal ultrasound than at pathological examination [12], and postmortem assessment may be limited or impossible because of fetal fragmentation following labor or termination of pregnancy. Moreover, classifications derived from postmortem data do not fully encompass the range of phenotypes observed prenatally [12].

As ultrasound remains the gold standard for prenatal diagnosis, there is a clear need for a classification framework based on sonographic rather than postmortem findings. Identification of prenatal phenotypes of complex ventral wall defects (VWDs) may contribute to a better understanding of their pathogenesis, improve diagnostic consistency, and enhance prenatal counselling, while also guiding future research directions.

In recent years, unsupervised data-driven methods, particularly hierarchical clustering, have been increasingly applied in modern medical research to address phenotypic heterogeneity and overlapping disease presentations. Hierarchical clustering is widely used in medical imaging, genomics, and machine-learning-based phenotype discovery, where it enables objective identification of complex patterns without requiring a priori assumptions regarding diagnostic categories or the number of underlying groups [13,14,15]. In prenatal imaging, such approaches offer particular value by allowing classification to emerge directly from observable features, reflecting real-world diagnostic practice and minimizing subjective interpretation and classification bias.

The aim of this study was to apply unsupervised hierarchical cluster analysis to prenatal ultrasound data in order to define objective, sonographically based phenotypes of complex ventral wall defects observed in the prenatal period.

## 2. Material and Methods

A retrospective study on fetuses with complex VWDs diagnosed in the ultrasound unit of the Department of the Obstetrics and Gynecology, prof. Witold Orlowski Public Teaching Hospital in Warsaw, Centre of Postgraduate Medical Education between 1 January 1997 and 31 December 2021 was performed. All ultrasound examinations were performed by specialists with expertise in fetal medicine using Sequoia™ 512 Ultrasound System, Siemens Medical Solutions USA, Inc., Mountain View, CA, USA. (until 2014) or Voluson™ E8 Expert Ultrasound System, GE Healthcare, Zipf, Austria —(from 2015) in accordance with the ISUOG practice guidelines on routine first- and mid-trimester scans [16,17,18].

The inclusion criteria comprised all fetuses with a prenatal ultrasound diagnosis of complex ventral wall defects evaluated during the study period. The electronic database was searched for cases with prenatally diagnosed ventral wall defects (VWDs). Exclusion criteria included simple omphalocele, gastroschisis, and conjoined twins.

Each sonographic record was independently reviewed by two authors using stored videoclips when available. Sonographic features were categorized as described below. In cases of uncertainty, the final classification was reached after detailed discussion and consensus.

All sonographic features were categorized as binary variables (0 if the feature was absent and 1 if the feature was present). Structural anomalies were classified into the following categories: ventral wall defects (VWDs), open neural tube defects (ONTD), spinal defects, limb anomalies, short umbilical cord, and other anomalies.

“Other defects” refers to anomalies not included among ventral wall defects, open neural tube defects, spinal defects, or limb anomalies. These additional anomalies were observed in 39 fetuses (24.4%) and included cardiac anomalies in 18 cases (11.3%) and non-cardiac anomalies in 21 cases (13.1%), such as renal anomalies and cleft lip and/or palate.

Ventral wall defects comprised thoracic wall, abdominal wall, and pelvic wall defects, either in isolation or in combination. Open neural tube defects included acrania, encephalocele, and spina bifida, either alone or in combination. Clubfoot, when associated with ONTD, was considered part of the ONTD spectrum.

Spinal defects were classified as kyphoscoliosis, spinal shortening, or a combination of both. Limb anomalies not belonging to the ONTD spectrum were subdivided into upper and lower limb anomalies. The umbilical cord was subjectively assessed as either absent or short.

Prenatal diagnosis in this study was based exclusively on ultrasound examination; fetal MRI was not performed in any case. The primary aim of the study was to define sonographically based prenatal phenotypes of complex ventral wall defects, and therefore only ultrasound-derived variables were used in the clustering analysis.

Given the life-limiting nature of complex ventral body wall abnormalities, invasive genetic testing was offered on an individual basis, taking into account the gestational age at diagnosis. Genetic testing was performed in two collaborating genetic laboratories.

Until 2016, conventional karyotyping was carried out following standard flask culture of amniocytes, long-term culture of chorionic villi, or standard lymphocyte culture. G-banding using Giemsa staining (GTG) was applied at the Genetic Department of the Institute of Psychiatry and Neurology in Warsaw.

From 2017 onward, chromosomal microarray analysis (CMA) was offered to all patients with fetal anomalies at the Institute of Mother and Child, Warsaw, Poland [19]. CMA was performed on DNA extracted from uncultured amniotic fluid or chorionic villi using the oligonucleotide array platform CytoSure Constitutional v3 (8 × 60 k) (Oxford Gene Technology), comprising approximately 60,000 probes across the genome. Data were analyzed using CytoSure™ Interpret Software, Oxford Gene Technology (OGT), Begbroke, Oxfordshire, UK.), providing an average resolution of 120 kb. Detected copy number variants were classified in accordance with the European guidelines for constitutional cytogenomic analysis. In all cases, routine karyotyping was additionally performed to exclude polyploidy and structural chromosomal abnormalities that are not detectable by CMA.

Statistical analysis was performed using STATA version 12 software (StataCorp LP), python libraries scikit-learn and eli5. Descriptive statistics were presented as means, medians, and percentages. Associations between categorical variables were assessed using the chi-square test or Fisher’s exact test, as appropriate, and logistic regression analysis was used to evaluate relationships between categorical outcomes.

Cluster analysis was conducted by an experienced data science researcher (MB). First, all data were manually verified by two independent sonographers (JB and AKC), and sonographic features were grouped into a coherent set based on medical expertise. Second, observations were clustered using an agglomerative hierarchical clustering algorithm. Multiple hyperparameters were tested, including distance metrics (Dice, cosine, and Euclidean), numbers of clusters (ranging from two to five), and linkage methods (average, complete, and Ward). The optimal combination of hyperparameters was selected based on the silhouette score of the clustering solution (range −1 to +1, with higher values indicating better cluster separation).

Finally, each cluster (phenotype) was characterized by its most discriminative features, based on feature importance values and overrepresentation or underrepresentation ratios (ranging from +1.0 for the most overrepresented features to −1.0 for the most underrepresented features). In conventional classification tasks, feature importance reflects the contribution of a given feature to assigning an observation to a specific class or cluster; however, such measures are often difficult to interpret and apply in clinical obstetric practice. Therefore, we placed particular emphasis on interpretability.

Feature importance in this study was defined as follows: feature importance was calculated on the outcome of logistic regression which was applied on the outcome of unsupervised clustering done earlier. Feature importance in logistic regression is determined by the magnitude and sign of the coefficients (β), indicating how much a feature changes the log-odds of the outcome, with larger absolute values meaning greater influence. Logistic regression was performed with scikit-learn library and feature importance was calculated with eli5 library.

Over-representation or under-representation ratios in this study were defined as follows:

Over/under-representation of feature *F* in cluster N = mean value of feature *F* in cluster N—mean value of feature *F* in all other clusters combined (excluding cluster N).

Because all features describing each observation were binary (1 if the feature was present and 0 if absent), averaging was mathematically appropriate. With this definition, over-representation or under-representation ratio represents the degree to which a given feature differentiates a specific cluster from the remaining clusters. When ranked in descending order, these values highlight the most overrepresented and therefore most discriminative features for each cluster.

The percentages represent the proportion of each anomaly within each cluster. For instance, the presence of spinal anomalies of any type across all five clusters at different percentages reflects their varying prevalence within each group. These differences indicate that spinal anomalies contribute to cluster differentiation, and more pronounced differences in their prevalence across clusters support better separation and stronger clustering performance, as the clustering method is based on maximizing differences in anomaly distributions among clusters.

Cluster analysis was selected because the primary aim of the study was exploratory, focusing on identifying natural phenotypic groupings among fetuses with complex ventral wall defects without predefined outcome labels. Agglomerative hierarchical clustering was chosen because it does not require a priori specification of the number of clusters and enables assessment of similarity relationships across multiple levels of granularity through dendrograms. This property is particularly advantageous for heterogeneous and relatively small clinical datasets, where the true number of underlying phenotypes is unknown and phenotypic boundaries are not clearly defined.

In contrast, k-means clustering requires predefining the number of clusters and assumes spherical, similarly sized clusters centered around a mean, assumptions that are often violated in clinical and imaging data characterized by high variability and overlapping features. Moreover, k-means is sensitive to initialization and outliers, and does not provide a hierarchical representation of relationships between clusters, limiting interpretability in exploratory medical research.

Supervised methods such as decision trees or neural networks were not applied, as they require labeled data and are primarily designed for predictive modeling rather than pattern discovery. Similarly, conventional data augmentation and cross-validation strategies are mainly applicable to supervised learning frameworks and were therefore not directly applicable in this unsupervised clustering context (see Supplementary Materials, Figures S1–S3.

In case of descriptive, retrospective studies institutional ethics committee permission is not necessary. Nevertheless, an internal bioethics committee approved the study design. All patients were informed and consented to use of their anonymized data for research purpose.

**Figure 1:**
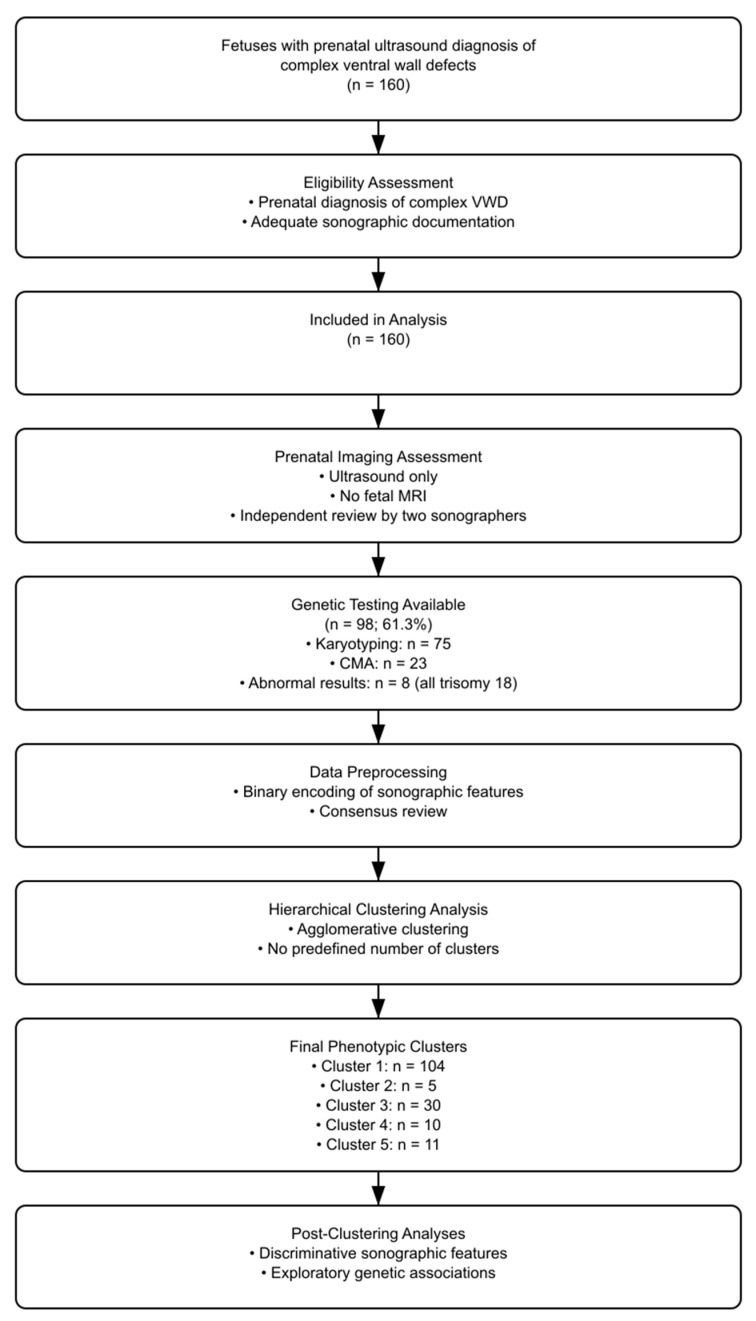
Study flow diagram.

The study flow is illustrated in Figure 1.

## 3. Results

A total of 160 fetuses with complex ventral wall defects were evaluated at our institution during the study period, including three fetuses from twin pregnancies discordant for the anomalies (one monochorionic monoamniotic, one monochorionic diamniotic, and one dichorionic twin pregnancy). The mean maternal age was 28.0 years (SD 5.9).

The mean and median gestational ages at diagnosis were 16 and 14 weeks, respectively. Ninety-one patients were referred before 14 weeks’ gestation, accounting for 56.9% of cases (91/160). Over the study period, the mean gestational age at diagnosis decreased from 16.6 weeks in 1997 to 14.1 weeks in 2021, corresponding to a reduction of approximately 0.31 weeks per year.

Ventral wall defects involved the abdominal wall in 150 cases, the thoracic wall in 42 cases, and the pelvic wall in 28 cases, either in isolation or in combination (thoracic *n* = 6; thoracoabdominal *n* = 33; thoracoabdominopelvic *n* = 3; abdominal *n* = 93; abdominopelvic *n* = 21; pelvic *n* = 4). Open neural tube defects were present in 58 fetuses, including acrania in 34 cases, encephalocele in 6 cases, and spina bifida in 28 cases. Complete craniorachischisis was observed in five cases, and acrania was associated with spina bifida in four cases (lumbosacral *n* = 3; cervical *n* = 1). One fetus presented with an occipital encephalocele and complete rachischisis.

Spinal defects were identified in 110 fetuses (68.8%), including kyphoscoliosis in 99 cases and spinal shortening in 34 cases. Limb anomalies were reported in 45 fetuses (28.1%), affecting the upper limbs in 18 cases and the lower limbs in 32 cases. Additional anomalies were present in 39 fetuses (24.4%), comprising cardiac anomalies in 18 cases (11.3%) and non-cardiac anomalies in 21 cases (13.1%). Amniotic bands were observed in seven cases (4.4%).

**Figure 2:**
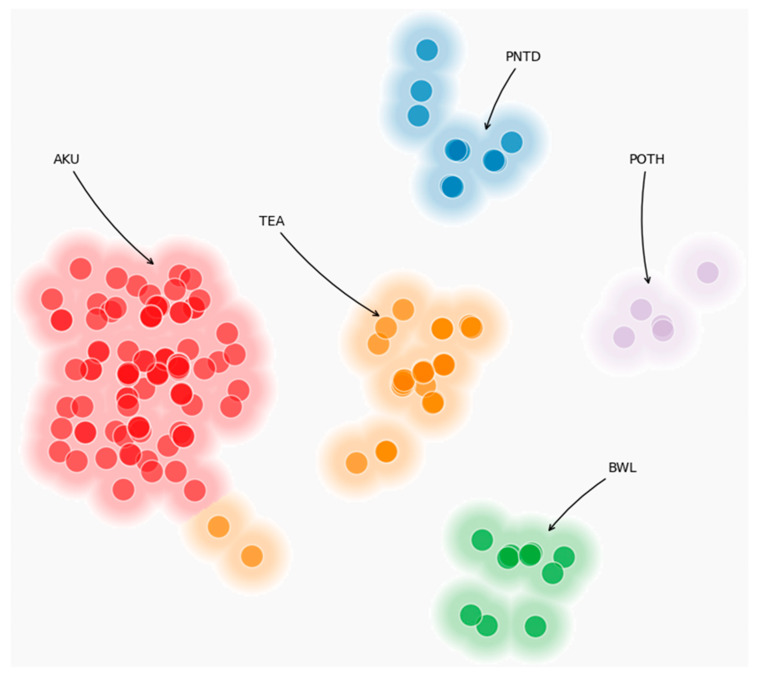
Five clusters of fetuses with ventral wall defect based on ultrasound features. Figure 1 represents a two-dimensional visualization of the clustering results. The *X*-axis and *Y*-axis do not correspond to specific clinical variables; instead, they represent the first two dimensions of a low-dimensional projection derived from the original high-dimensional feature space used for clustering. These dimensions are unitless and are intended solely to facilitate visual interpretation of the relative similarity between observations. Each point corresponds to a single fetus, and the spatial proximity between points reflects similarity in the underlying binary sonographic feature set: observations plotted closer together are more similar, whereas those farther apart are more dissimilar. The colored regions illustrate the clusters identified by the agglomerative hierarchical clustering algorithm, with labels indicating the predominant phenotypic patterns within each cluster. The axes represent abstract projection dimensions used for visualization purposes only and should not be interpreted as individual clinical or anatomical variables. Abbreviations: AKU; A—abdomen, K—kyphoscoliosis U—short umbilical cord; POTH; P—pelvis, O—other; TEA; T—thorax, E—ectopia cordis, A—acrania; BWL; Body wall, L—limbs; PNTD; P—pelvis, NTD—neural tube defect.

Using agglomerative hierarchical clustering, we identified five groups of fetuses with differing numbers of observations (cluster 1, *n* = 104; cluster 2, *n* = 5; cluster 3, *n* = 30; cluster 4, *n* = 10; cluster 5, *n* = 11), as illustrated in Figure 2.

Cluster 1 was characterized by abdominal wall defects, kyphoscoliosis, and a short umbilical cord (abbreviation AKU; A = abdomen, K = kyphoscoliosis, U = short umbilical cord). Cluster 2 was characterized by pelvic wall defects and anomalies of organs other than the neural tube, spine, and limbs (abbreviation POTH; P = pelvis, O = other). Cluster 3 was characterized by thoracic wall defects, ectopia cordis, and acrania (abbreviation TEA; T = thorax, E = ectopia cordis, A = acrania). Cluster 4 was characterized by abdominopelvic wall defects and limb anomalies (abbreviation BWL; B = body wall, L = limbs). Cluster 5 was characterized by pelvic wall defects associated with neural tube defects (abbreviation PNTD; P = pelvis, NTD = neural tube defect).

**Figure 3:**
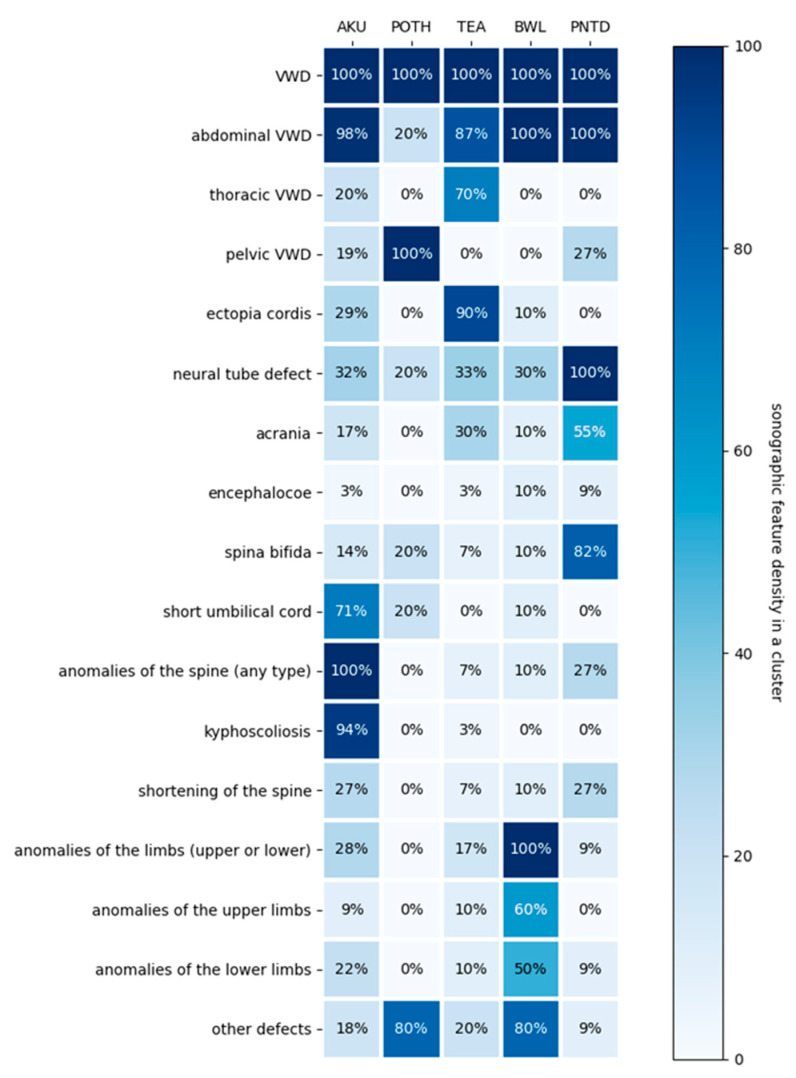
Sonographic feature density in clusters. The percentages represent the proportion of cases within each cluster that present a specific anomaly. Larger differences across clusters indicate stronger separation and better clustering performance.

**Figure 4:**
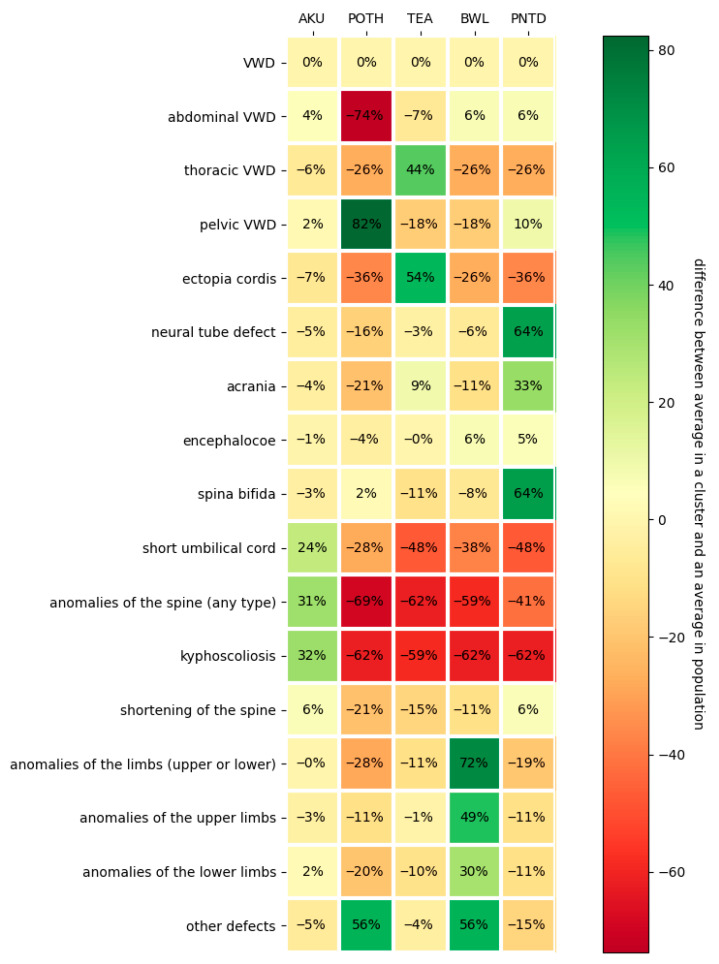
Difference between the average feature frequency in a cluster and the average in a population. Larger differences across clusters indicate stronger separation and better clustering performance.

The silhouette score of the clustering model was 0.3285, indicating a reasonable degree of separation between clusters. Sonographic findings according to cluster assignment are summarized in Table 1 and Table 2 and illustrated in Figure 3 and Figure 4. The most discriminative features for each cluster, expressed as feature importance values, were as follows: kyphoscoliosis for cluster 1 (0.924), pelvic wall defect for cluster 2 (0.852), ectopia cordis for cluster 3 (0.662), limb anomalies for cluster 4 (0.767), and spina bifida for cluster 5 (0.691). Further details are provided in Table 2.

Genetic testing results were available in 98 of 160 fetuses (61.3%). Until 2016, conventional karyotyping was performed (*n* = 75), and from 2017 onward, chromosomal microarray analysis (CMA) was offered to all patients with fetal anomalies (*n* = 23). Overall, abnormal genetic results were identified in 8 of 98 tested fetuses (8.2%), and in all cases the abnormality was trisomy 18. In additional two cases CMA revealed CNVs in chromosome 16 (arr[GRCh37] 16p13.11(14910213_16194575)x3 pat; arr[GRCh37] 16p11.2(28843754_29031071)x1), which were categorised as variants of unknown significance (VUS).

When genetic findings were analyzed in relation to the identified phenotypic clusters, trisomy 18 was observed in all clusters except cluster 2. The frequency of abnormal genetic results varied across clusters (3.2%, 0.0%, 10.5%, 28.6%, and 33.3% in clusters 1–5, respectively), with a statistically significant overrepresentation in clusters 4 and 5 (cluster 4: OR 15.0, 95% CI 1.652–136.172, *p* = 0.016; cluster 5: OR 12.0, 95% CI 1.381–104.251, *p* = 0.024) (Figure 4).

## 4. Discussion

To date, various criteria have been proposed to classify complex malformations of the fetal ventral wall [4,5,6,10,20]. Nevertheless, despite the efforts of many authors, a unified classification and a clear pathological explanation of complex ventral wall defects (VWDs) are still lacking. We therefore aimed to apply an objective approach to analyze the heterogeneity of these fetuses.

In contrast to previous studies with heterogeneous inclusion criteria and reliance on postmortem findings, we included all fetuses with sonographically detected VWDs other than simple gastroschisis and simple omphalocele. These cases were clustered according to the level of the defect and associated anomalies. Under the assumption that all fetuses with VWDs initially belong to a single group, agglomerative clustering was used to group cases based on shared associated anomalies, thereby identifying more homogeneous subgroups and providing new insight into the underlying pathologies.

Our study confirms that complex malformations of the fetal ventral body wall do not represent a single entity but rather comprise distinct phenotypes with overlapping features. The incorporation of mathematical clustering methods into prenatal sonography allowed for an objective classification of these malformations into different phenotypes and reduced the bias that is inevitable with purely subjective assessment of sonographic features.

Clustering algorithms differ substantially in how they define a cluster and in the strategies used to group observations efficiently [13,21,22,23]. The selection of appropriate parameters—such as the expected number of clusters, density thresholds, and the distance metric used to quantify similarity—depends on the characteristics of the specific dataset. In the present study, data preprocessing was performed individually for each observation by two independent sonographers to ensure consistency and reduce observer bias.

We employed agglomerative hierarchical clustering, an unsupervised approach that begins with each observation as a single cluster and progressively merges the most similar observations, in contrast to divisive methods that start with the complete dataset and iteratively partition it. This method is based on the principle that objects in close proximity within the feature space are more closely related than those farther apart. Cluster merging is guided by a measure of dissimilarity between sets of observations, calculated using Euclidean distances between individual observations in combination with a linkage criterion that defines inter-cluster dissimilarity as a function of pairwise distances. In this framework, clusters are characterized by the maximum distance required to connect their constituent elements.

Cluster quality was assessed using the silhouette value, which quantifies how similar an observation is to its own cluster (cohesion) compared with other clusters (separation). Silhouette values range from −1 to +1, with higher values indicating better assignment of observations to their respective clusters [24].

Agglomerative hierarchical clustering was selected over alternative approaches, such as centroid-based or density-based methods, because it provides a complete hierarchical representation of the data structure in the form of dendrograms. This enables exploration of relationships between clusters across multiple levels of granularity and does not require an a priori assumption regarding the number of clusters. These characteristics are particularly advantageous in contexts where the true number of underlying phenotypic groups is unknown or conceptually ambiguous, as further discussed in the Supplement.

Using this approach, we identified five distinct groups of fetuses with complex ventral wall defects (VWDs). The largest group (cluster 1, AKU) comprised 104 fetuses and exhibited features consistent with previous definitions of body stalk anomaly, including severe abdominal wall defects, kyphoscoliosis, and a short umbilical cord [6]. In over 60% of fetuses in this cluster, the ventral wall defect was confined to the abdominal level. Notably, all but two fetuses with a short umbilical cord in the entire study population were assigned to this cluster.

However, the most discriminative feature for this group was kyphoscoliosis, with an importance value of 0.924, whereas the importance of a short umbilical cord was lower (0.676). Only one fetus with kyphoscoliosis was assigned to a different cluster, namely cluster 3 (TEA), which included 30 fetuses and was characterized by thoracic or thoracoabdominal wall defects, resembling the pentalogy of Cantrell [9]. As shown in Figure 1, clusters 1 and 3 were closely related, and some fetuses in cluster 3 shared features with those in cluster 1.

Cluster 4 (BWL) consisted of 10 fetuses with abdominopelvic wall defects associated with limb anomalies and cardiac defects. Cluster 5 (POTH) comprised 11 fetuses with abdominal or abdominopelvic VWDs combined with neural tube defects and spinal shortening. The smallest group, cluster 2, included five fetuses and was characterized by purely pelvic or abdominopelvic wall defects and anomalies of organs other than the neural tube, spine, and limbs, most of which were non-cardiac.

Internal and external cluster coherence is illustrated in Figure 1, while the defining features of each cluster are summarized in Table 1 and Table 2. The silhouette score of the clustering model was 0.329, indicating that the model provides a reasonable representation of similarities and dissimilarities among fetuses with complex VWDs.

Several issues related to the interpretation of our results merit discussion. Some sonographic features, such as ectopia cordis and thoracoabdominal ventral wall defects, or neural tube defects and acrania, are not independent. We did not assign different weights to non-independent features, which may have led to some redundancy when interpreting Figure 1, Figure 2 and Figure 3, as both features contributed strongly to the phenotype. In addition, features with very high prevalence may lack discriminative value. This was the case for abdominal-level ventral wall defects, which were present in nearly 100% of cases in clusters 1, 3, 4, and 5 and therefore did not contribute to cluster differentiation.

A major strength of our study is that, in contrast to previous reports, it is based exclusively on prenatal sonographic findings. Although the application of clustering methods in prenatal diagnosis is not entirely novel—Rittler et al. used cluster analysis to distinguish between body stalk anomaly and amniotic band syndrome [25]—their study relied on postmortem examinations and was limited to fetuses and neonates diagnosed with limb body wall complex (LBWC) or amniotic band syndrome (ABS). In contrast, we included all fetuses with complex ventral wall defects, thereby minimizing bias introduced by pre-established diagnoses. Moreover, in the study by Rittler et al., cases were identified based on diagnostic codes assigned by attending pediatricians [25]. Given the long study period (1967–2013), this approach is likely to have introduced additional bias, particularly since widely accepted definitions of ABS and LBWC were first described in the late 1970s [4,5,8], whereas the analyzed study period began in 1967. In our study, only prenatal sonographic reports were analyzed, enabling a classification more appropriate for the prenatal setting.

Consistent with our previous observations [12], amniotic bands were much less common in our cohort than reported by other authors. We therefore suggest that amniotic bands should not be used as a discriminative feature when classifying complex ventral wall defects. Nevertheless, all cases with amniotic bands in our study were assigned to cluster 1 (AKU).

Rittler et al. proposed that only a specific type of limb reduction defect, namely amelia, should be considered a criterion for LBWC [25]. Our data do not allow us to draw a similar conclusion, as limb reduction defects were not subclassified in our cohort. Although the majority of limb anomalies in our study population (64.4%, 29/45) occurred in cluster 1 (AKU), limb anomalies were the most discriminative feature for cluster 4 (BWL), with an importance value of 0.767.

The frequency of abnormal genetic findings varied across clusters, with a statistically significant overrepresentation in clusters 4 and 5. Notably, cluster 5 was predominantly composed of fetuses with neural tube defects, mainly acrania and spina bifida with spinal shortening. This finding underscores the importance of offering invasive genetic testing even in cases traditionally considered uniformly lethal.

However, genetic data were not incorporated into the clustering analysis because genetic testing was not performed uniformly across the cohort and the number of abnormal results was limited, restricting the robustness of genotype–phenotype correlations. These findings should therefore be interpreted with caution.

In addition, chromosomal microarray analysis identified copy number variants involving chromosome 16 in two fetuses from cluster 1, both classified as variants of uncertain significance. Previous reports have described maternal uniparental disomy of chromosome 16 in association with limb body wall complex or body stalk anomaly, although such findings are typically confined to the placenta. Further studies, ideally incorporating advanced molecular techniques such as whole-exome or whole-genome sequencing, are needed to clarify the potential role of these findings in the pathogenesis of complex ventral wall defects.

Overall, our results support offering invasive prenatal genetic testing in cases of complex ventral wall defects regardless of phenotype and highlight the importance of comprehensive prenatal counseling in these pregnancies.

The main limitation of our study is its retrospective design. However, given the rarity of complex ventral wall defects, conducting a prospective study with a comparable sample size would be extremely challenging. All examinations were performed or supervised by a single experienced sonographer using a strict evaluation protocol consistent with ISUOG guidelines [16,17,18]. To ensure the reliability of our findings, each sonographic record was independently reviewed and manually verified by two authors (JB and AKC).

Another potential limitation is the variability in gestational age at the time of evaluation. Complex malformations of the fetal ventral body wall are typically readily identifiable in the first or early second trimester. Because these anomalies are life-limiting, a high rate of pregnancy termination has been reported, which may have resulted in underdiagnosis of certain structural defects that are more likely to be detected later in gestation.

## 5. Conclusions

In conclusion, complex malformations of the fetal ventral wall are associated with a wide and heterogeneous spectrum of additional anomalies. Using hierarchical clustering, we identified five distinct phenotypic groups of complex ventral wall defects, namely AKU, POTH, TEA, BWL, and PTND. This objective, data-driven approach highlights that these conditions do not represent a single pathological entity but rather comprise several overlapping phenotypes with shared and distinguishing features.

The integration of advanced molecular genetic techniques, including exome and genome sequencing, has the potential to further improve our understanding of the underlying developmental mechanisms and pathogenetic pathways involved in complex ventral wall defects. Such approaches may help to refine phenotype–genotype correlations, improve classification, and ultimately support more accurate prenatal diagnosis, prognostication, and counselling for affected pregnancies.

## Figures and Tables

**Table 1:** Structural anomalies observed in the study group with regard to cluster.

Defect	1 (AKU) (*n* = 104)	2 (POTH) (*n* = 5)	3 (TEA) (*n* = 30)	4 (BWL) (*n* = 10)	5 (PTND) (*n* = 11)
Ventral wall thoracic thoracoabdominal thoracoabdominopelvic abdominal abdominopelvic pelvic	104 (100.0%) 2 (1.9%) 16 (15.4%) 3 (2.9%) 66 (63.5%) 17 (16.4%) 0 (0.0%)	5 (100.0%) 0 (0.0%) 0 (0.0%) 0 (0.0%) 0 (0.0%) 1 (20.0%) 4 (80.0%)	30 (100.0%) 4 (13.3%) 17 (56.7%) 0 (0.0%) 9 (30.0%) 0 (0.0%) 0 (0.0%)	10 (100.0%) 0 (0.0%) 0 (0.0%) 0 (0.0%) 0 (0.0%) 10 (100.0%) 0 (0.0%)	11 (100.0%) 0 (0.0%) 0 (0.0%) 0 (0.0%) 8 (72.7%) 3 (27.3%) 0 (0.0%)
Neural tube acrania encephalocoele spina bifida	33 (31.7%) 18 (17.3%) 3 (2.9%) 15 (14.4%)	1 (20.0%) 0 (0.0%) 0 (0.0%) 1 (20.0%)	10 (33.3%) 9 (30.0%) 1 (3.3%) 2 (6.7%)	3 (30.0%) 1 (10.0%) 1 (10.0%) 1 (10.0%)	11 (100.0%) 6 (54.6%) 1 (9.1%) 9 (81.8%)
Spine Kyphoscoliosis Shortening of the spine	104 (100.0%) 98 (94.2%) 28 (26.9%)	0 (0.0%) 0 (0.0%) 0 (0.0%)	2 (6.7%) 1 (3.3%) 1 (3.3%)	1 (10.0%) 0 (0.0%) 1 (10.0%)	3 (27.3%) 0 (0.0%) 3 (27.3%)
Limbs Upper limbs Lower limbs	29 (27.9%) 9 (8.7%) 23 (22.1%)	0 (0.0%) 0 (0.0%) 0 (0.0%)	5 (16.7%) 3 (10.0%) 3 (10.0%)	10 (100.0%) 6 (60.0%) 5 (50.0%)	1 (9.1%) 0 (0.0%) 1 (9.1%)
Short umbilical cord	74 (71.2%)	1 (20.0%)	0 (0.0%)	1 (10.0%)	0 (0.0%)
Other anomalies (cardiac)	7 (6.7%)	1 (20.0%)	1 (3.3%)	7 (70.0%)	1 (9.1%)
Other anomalies (non- cardiac)	15 (14.4%)	3 (60.0%)	6 (20.0%)	5 (20.0%)	0 (0.0%)

**Table 2:** The most common and the discriminative features of each cluster.

Cluster	Abbreviation	*n*	Predominant Ventral Wall Defect	The Most Common Additional Features (>50%)	The Most Discriminative Sonographic Features	Feature Importance
1	AKU	*n* = 104	abdominal	kyphoscoliosis short cord	kyphoscoliosis short umbilical cord abdominal wall defect	0.924 0.676 0.124
2	POTH	*n* = 5	pelvic	other defects (non-cardiac)	pelvic wall defect other abnormalities	0.852 0.581
3	TEA	*n* = 30	thoracoabdominal		ectopia cordis thoracic wall defect acrania	0.662 0.538 0.108
4	BWL	*n* = 10	abdominopelvic	limbs other (cardiac)	defects of the limbs other abnormalities	0.767 0.600
5	PNTD	*n* = 11	abdominal	neural tube	spina bifida any neural tube defect acrania pelvic wall defect	0.691 0.685 0.358 0.105

## Data Availability

Data available on request from the authors.

## Supplementary Materials

The following supporting information can be downloaded at: https://www.mdpi.com/article/10.3390/jcm15041343/s1, Figure S1: Schematic illustration of hierarchical clustering, in which each observation initially forms a separate cluster and the two most similar clusters are iteratively identified and merged until a single hierarchy is formed; Figure S2: Dendrogram representing the hierarchical structure and relationships among the identified clusters; Figure S3: Interpretation of a dendrogram in hierarchical clustering, where no prior assumption is made regarding the number of clusters. Cluster membership is defined by applying a horizontal cut to the dendrogram, with each branch below the cut representing a distinct cluster, while preserving information on relationships between subclusters and allowing adjustment of clustering granularity.
