# Supplementary material for: Integrating Mathematics into Prenatal Diagnosis—Different Phenotypes of Complex Ventral Wall Malformations Determined by Hierarchical Clustering
Source: J Clin Med. 2026 Feb 8;15(4):1343. doi: 10.3390/jcm15041343 (PMC12941328; doi:10.3390/jcm15041343)
Supplement: Supplementary file 1 [file jcm-15-01343-s001.zip › jcm-3942053-supplementary.pdf]

Supplement – Agglomerative Hierarchical Clustering

Agglomerative Hierarchical Clustering is one of the many algorithms in broad category of cluster analysis (clustering).

Cluster analysis or clustering is the task of grouping a set of objects in such a way that objects in the same group (called a cluster) are more similar (in some sense) to each other than to those in other groups (clusters).

Cluster analysis itself is not one specific algorithm, but the general task to be solved. It can be achieved by various algorithms that differ significantly in their understanding of what constitutes a cluster and how to efficiently find them.

Classes of cluster analysis algorithms

The notion of a "cluster" cannot be precisely defined, which is one of the reasons why there are so many clustering algorithms. The notion of a cluster, as found by different algorithms, varies significantly in its properties. Understanding these "cluster models" is key to understanding the differences between the various algorithms. Typical cluster models include:

- Connectivity models: for example, hierarchical clustering builds models based on distance connectivity.
- Centroid models: for example, the k-means algorithm represents each cluster by a single mean vector.
- Distribution models: clusters are modeled using statistical distributions, such as multivariate normal distributions used by the expectation-maximization algorithm.
- Density models: for example, DBSCAN and OPTICS defines clusters as connected dense regions in the data space.

Hierarchical clustering

Connectivity-based clustering, also known as hierarchical clustering, is based on the core idea of objects being more related to nearby objects than to objects farther away. These algorithms connect "objects" to form "clusters" based on their distance. A cluster can be described largely by the maximum distance needed to connect parts of the cluster. At different distances, different clusters will form, which can be represented using a dendrogram, which explains where the common name "hierarchical clustering" comes from: these algorithms do not provide a single partitioning of the data set, but instead provide an extensive hierarchy of clusters that merge with each other at certain distances. In a dendrogram, the y-axis marks the distance at which the clusters merge, while the objects are placed along the x-axis such that the clusters don't mix.

How hierarchical clustering works

Hierarchical clustering starts by treating each observation as a separate cluster. Then, it repeatedly executes the following two steps: (1) identify the two clusters that are closest together, and (2) merge the two most similar clusters. This iterative process continues until all the clusters are merged together. This is illustrated in the diagrams below.

Identify the two clusters that are **closest** together	Merge the two closest clusters

Figure S1

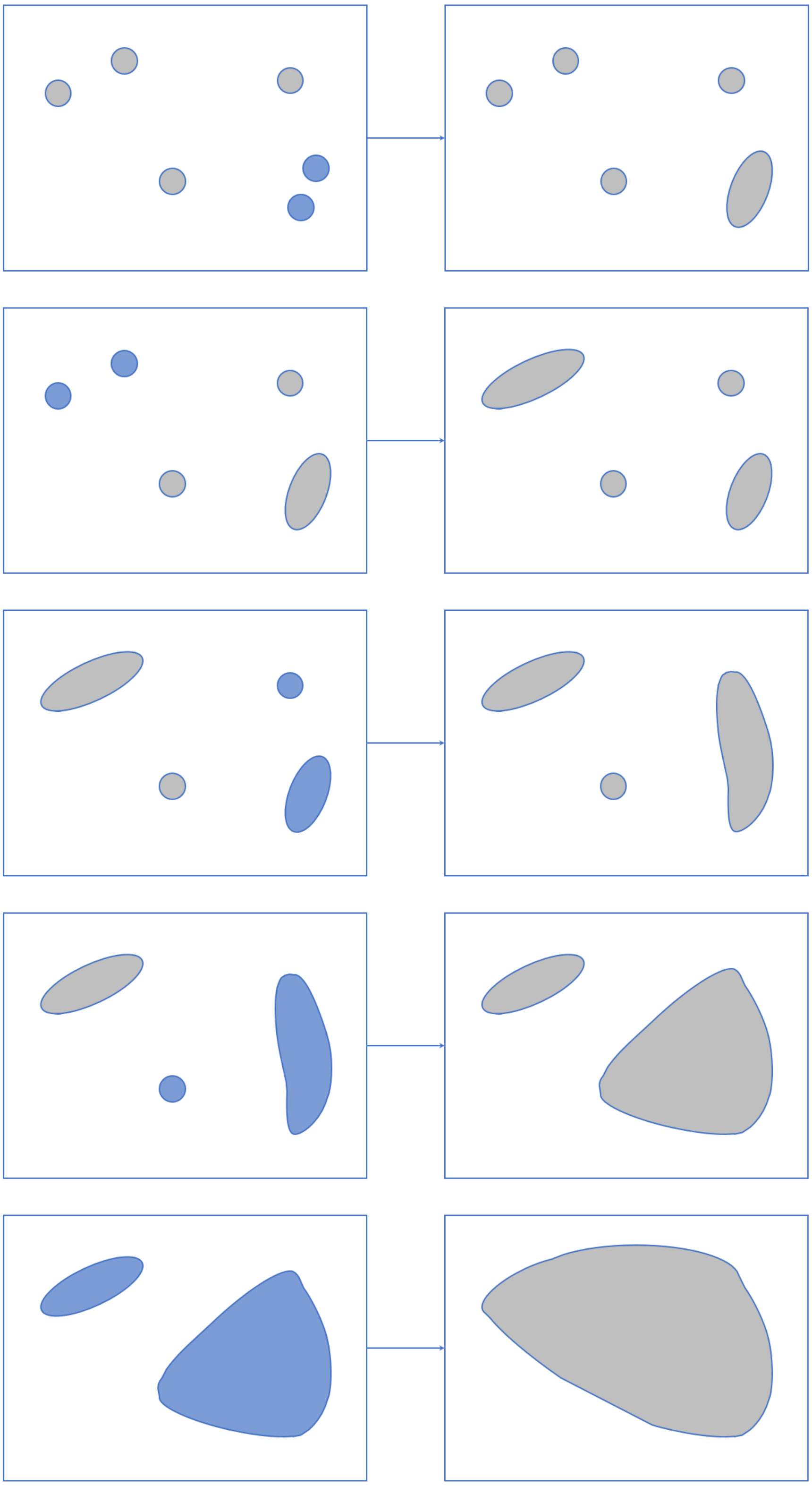

The main output of Hierarchical Clustering is a dendrogram, which shows the hierarchical relationship between the clusters:

Figure S2

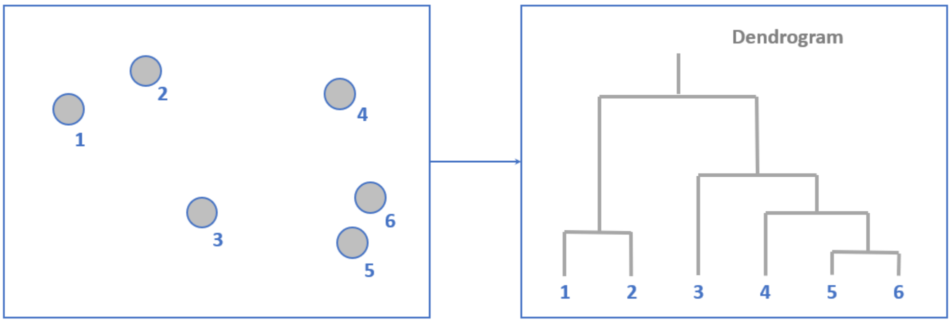

Measures of distance (similarity)

In the example above, the distance between two clusters has been computed based on the length of the straight line drawn from one cluster to another. This is commonly referred to as the Euclidean distance. Many other distance metrics have been developed.

The choice of distance metric should be made based on theoretical concerns from the domain of study. That is, a distance metric needs to define similarity in a way that is sensible for the field of study. For example, if clustering crime sites in a city, city block distance may be appropriate. Or, better yet, the time taken to travel between each location. Where there is no theoretical justification for an alternative, the Euclidean should generally be preferred, as it is usually the appropriate measure of distance in the physical world.

Linkage criteria

After selecting a distance metric, it is necessary to determine from where distance is computed. For example, it can be computed between the two most similar parts of a cluster (single-linkage), the two least similar bits of a cluster (complete-linkage), the center of the clusters (mean or average-linkage), or some other criterion. Many linkage criteria have been developed.

As with distance metrics, the choice of linkage criteria should be made based on theoretical considerations from the domain of application. A key theoretical issue is what causes variation. For example, in archeology, we expect variation to occur through innovation and natural resources, so working out if two groups of artifacts are similar may make sense based on identifying the most similar members of the cluster.

Where there are no clear theoretical justifications for the choice of linkage criteria, Ward's method is the sensible default. This method works out which observations to group based on reducing the sum of squared distances of each observation from the average observation in a cluster.

Number of clusters

In hierarchical clustering, while constructing the dendrogram, we do not keep any assumption on the number of clusters. Once the dendrogram has been constructed, we slice this structure horizontally. All the resulting child branches formed below the horizontal cut represent an individual cluster at the highest level in your system and it defines the associated cluster membership for each data sample. Note we are saying it as the highest level because even after you have created the clusters, you are still aware of what would be the relationship within the subsequent subclusters that can still be formed and you always have an option to increase/decrease the granularity level of clustering.

Dendrograms however do not do proper justice to understand how the clusters will look like after you place the horizontal cut. You have to individually mark the data points in a feature vector space with the resulting cluster indexes to visually see the effect of clustering.

The next question to ponder is where should you place the horizontal cut. The location of slicing can either be decided visually or even with the opinion that you desire to have a minimum distance of 'y' (the location of cut in the y-axis) between your clusters.

Figure S3

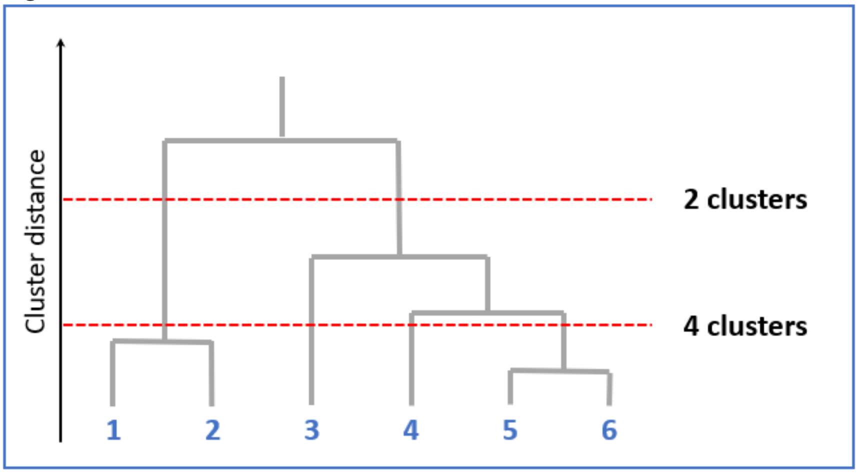

Construction of dendrogram

There are two ways of constructing dendrogram. One way to construct it is by bottoms-up where you start from the bottom and keep merging the individual data points and subclusters and go all the way to the top. This is known as agglomerative clustering.

The other alternative is the opposite procedure of top-down in which you start by considering the entire system as one cluster and then keep sub clustering it until you reach individual data samples. This process is known as divisive clustering. Each of these methods has separate algorithms to achieve its objectives.
